# A systematic review of school-based sexual health interventions to prevent STI/HIV in sub-Saharan Africa

**DOI:** 10.1186/1471-2458-8-4

**Published:** 2008-01-07

**Authors:** Virginia A Paul-Ebhohimhen, Amudha Poobalan, Edwin R van Teijlingen

**Affiliations:** 1 Health Services Research Unit, University of Aberdeen, Aberdeen, UK; 2 Department of Public Health, University of Aberdeen, Aberdeen, UK

## Abstract

**Background:**

The HIV/AIDS epidemic remains of global significance and there is a need to target (a) the adolescent age-groups in which most new infections occur; and (b) sub-Saharan Africa where the greatest burden of the epidemic lies. A focused systematic review of school-based sexual health interventions in sub-Saharan Africa to prevent HIV/AIDS and Sexually Transmitted Infections (STI) in this age group was therefore conducted.

**Methods:**

Searches were conducted in Medline, Embase, Cinahl and PsychINFO according to agreed *a priori *criteria for studies published between 1986 and 2006. Further searches were conducted in UNAIDS and WHO (World Health Organization) websites, and 'Google'. Relevant journals were hand-searched and references cited in identified articles were followed up.

Data extraction and quality assessment was carried out on studies selected for full text appraisal, and results were analysed and presented in narrative format.

**Results:**

Some 1,020 possible titles and abstracts were found, 23 full text articles were critically appraised, and 12 articles (10 studies) reviewed, reflecting the paucity of published studies conducted relative to the magnitude of the HIV epidemic in sub-Saharan Africa. Knowledge and attitude-related outcomes were the most associated with statistically significant change. Behavioural intentions were more difficult to change and actual behaviour change was least likely to occur. Behaviour change in favour of abstinence and condom use appeared to be greatly influenced by pre-intervention sexual history.

**Conclusion:**

There is a great need in sub-Saharan Africa for well-evaluated and effective school-based sexual health interventions.

## Background

HIV (human immunodefinciency virus) and AIDS (acquired immune deficiency syndrome) have reached epidemic proportions in most parts of sub-Saharan Africa by the end of the last century. 20.8 million HIV/AIDS cases recorded in this region in 1997, was equivalent to two-thirds of the global total for that year, a pattern which has been sustained for almost a decade [1]. Current estimates are that half of all new cases of HIV infection occur in people under the age of 25 years [1] and that 80% of AIDS cases worldwide are in those aged between 15 and 24, three-quarters of whom live in sub-Saharan Africa [2,3].

The natural history of HIV with a long latency period between acquired infection and manifestation of AIDS with the above figures implies that the greater percentage of HIV infections have been acquired during adolescence, to manifest as AIDS a decade later [4]. Furthermore, recent reassessment of HIV and sexually transmitted infections (STI) has shown HIV to alter the pattern of STI, the presence of STI to be associated with increased risk for acquiring HIV infection, and the concurrent existence of both to be associated with increased infectivity of an individual to sexual partners [5,6].

Targeting the right population at the right time such as making high-risk groups the priority for preventive interventions at the earliest stage of the epidemic has been identified as an important strategy for HIV/AIDS [7]. Therefore, in sub-Saharan Africa, where young persons now constitute the main pool sustaining the epidemic, the role of youth specific interventions aimed at preventing STI/HIV/AIDS cannot be overestimated.

In conducting a focused systematic review [8] of interventions to prevent HIV/STI in adolescents in sub-Saharan Africa, school-based interventions were chosen because of the practicality of the school setting in executing interventions to reach this age group and documented evidence supporting the perceived credibility by young people of interventions implemented in schools.

More specifically, with the threatened existence to traditional institutions like the age group/age grade and strong extended family network in many African communities, being replaced by formal western style of education, African pupils now expect to get correct sexual information in the school setting [9,10].

Unlike three similar reviews [11-13] conducted previously, our review is different in a number of ways. Firstly, it was focused geographically to sub-Saharan Africa where the main burden of the AIDS epidemic lies, rather than the entire developing world as done by Magnussen *et al. *[12], and Speizer *et al*. [13]. This was to reduce heterogeneity and increase the validity of the review findings to the population represented by participants in the review. Secondly, our review excluded programmes involving other setting (e.g. community component) as done by Brieger *et al. *[14]. This was to allow for more objective attributions to the effect of setting, as well as reducing studies' heterogeneity. Thirdly, unlike the similar 2004 review by Gallant and Maticka-Tyndale [11], we included only studies with control groups to ensure the highest quality studies (studies with control group have been shown not to differ systematically in estimating magnitude of effects whether randomised or non-randomised [15], and are ranked highest in hierarchy of study types [16]), and to reduce heterogeneity (based on initial consideration of possible meta-analysis. Fourthly, our review is more up-to-date, and more stringent, for example, our review excluded one of the eight studies that Gallant and Maticka-Tyndale had included, on grounds of low attrition [17]. Whilst we included one study [18] which Gallant and Maticka-Tyndale [11] had 'missed'.

## Methods

The first author developed the research protocol for this systematic review and established the *a priori *criteria for studies under guidance from the third author.

A pilot search strategy with search terms around the main subject themes i.e. 'studies', 'HIV and STI', 'school-based interventions' and 'limiting to Africa and to the period from 1986–2006', was developed by the first author, under guidance from the second author, and conducted in Medline (see Table 1) before being adapted for use in Embase and Cinahl and citation alerts set up in these databases during the period of the study (April to July 2006). In Psych INFO (1985–2006) the 'change database and re-run search strategy' command was used with the Medline search strategy (Table 1), followed by exclusion of the term 'exp Africa' in the final combination of themes. Commonly cited journals (such as *AIDS*, *AIDS Care *and *Journal of Adolescent Health*) were hand searched and websites of the WHO (World Health Organization), UNAIDS (United Nations Programme on HIV/AIDS) and 'Google' were browsed for relevant publications and reports. References of identified articles were also scanned for secondary references and all possible references exported into Refworks bibliographic management software. No language restrictions were applied to any electronic bibliographic searches.

**Table 1:** Search Strategy conducted in MEDLINE, EMBASE and CINAHL

Ovid MEDLINE(R) **1966 to April Week 2 2006**	EMBASE **1988 to 2006 Week 16**	CINAHL – Cumulative Index to Nursing, Allied Health Literature **1982 to April Week 3 2006**
Exp Randomized Controlled Trials/	Randomized Controlled Trial/	exp Clinical Trials/
Exp Random Allocation/	exp Randomization/	exp Random Assignment/
Exp Controlled Clinical Trials/	Clinical Trial/	exp Double-Blind Studies/
Exp Clinical Trials/	exp CONTROL/	exp Single-Blind Studies/
Exp Double-Blind Method/	exp Control Group/	exp Comparative Studies/
Exp Single-Blind Method/	exp Case Control Study/	exp Prospective Studies/
Exp Evaluation Studies/	Double Blind Procedure/	exp Concurrent Prospective Studies/
Exp Intervention Studies/	Single Blind Procedure/	exp Pilot Studies/
Exp Cohort Studies/	exp Comparative Study/	exp Validation Studies/
Exp Prospective Studies/	exp Prospective Study/	exp INTERVENTION TRIALS/
Exp Longitudinal Studies/	exp Longitudinal Study/	random$.tw.
Exp Follow-Up Studies/	exp Pilot Study/	(intervention$ adj5 stud$).tw.
Exp Epidemiologic Studies/	exp Evaluation/	(random$ or alloc$ or assign$).ti, ab.
Exp Multicenter Studies/	exp "Types of Study"/	exp Preventive Trials/
validation studies.tw.	exp Cohort Analysis/	or/1–14
random$.tw.	exp Follow Up/	exp SCHOOLS/
(intervention$ adj5 stud$).tw.	Multicenter Study/	exp Schools, Elementary/
(random$ or alloc$ or assign$).ti, ab.	Validation Process/	exp Schools, Middle/
or/1–18	random$.tw.	exp Schools, Secondary/
Exp Schools/	(intervention$ adj5 stud$).tw.	exp students, high school/
Exp Health Promotion/	(random$ or alloc$ or assign$).ti, ab.	exp Health Education/
Exp Health Education/	or/1–21	exp Health Promotion/
education.ti.	exp SCHOOL/	exp Sex Education/
Exp Sex Education/	exp Primary School/	exp School Health Education/
teaching.ti.	exp High School/	exp Student Health Education/
(school$ adj5 sex$ adj5 (educat$ or	exp School Health Service/	exp HIV Education/
promot$ or interven$ or teach$)).tw.	exp Health Promotion/	education.ti.
or/20–26	exp Health Education/	exp Safe Sex/
Exp Africa/	education.ti.	(school$ adj5 sex$ adj5 (educat$ or promot$ or interven$ or teach$)).tw.
Exp Sexually Transmitted Diseases/	exp Sexual Education/	or/16–28
Exp HIV/	Education Program/	exp "Africa South of the Sahara"/
Exp HIV Infections/	teaching.ti.	exp Sexually Transmitted Diseases/
Exp Sexual Behavior/	(school$ adj5 sex$ adj5 (educat$ or promot$ or interven$ or teach$)).tw.	exp CHLAMYDIA INFECTIONS/
Exp Unsafe Sex/	or/23–33	exp CHLAMYDIA/
Exp Gonorrhea/	exp AFRICA/	exp Chlamydia Trachomatis/
Exp Chlamydia/	exp "Africa South of the Sahara"/	exp GONORRHEA/
(sex$ adj3 behaviour$).tw.	35 or 36	exp Human Immunodeficiency Virus/
(sex$ adj3 transmit$ adj3 (disease$ or infection$)).tw.	exp Sexually Transmitted Disease/	exp HIV Infections/
or/29–37	exp Human Immunodeficiency Virus	exp Acquired Immunodeficiency Syndrome/
19 and 27 and 28 and 38	Infection/	(sex$ adj3 transmit$ adj3 (disease$ or infection$)).tw.
limit 39 to yr = "1986 – 2006"	exp CHLAMYDIASIS/	or/32–40
limit 40 to "all child (0 to 18 years)"	exp GONORRHEA/	15 and 30 and 31 and 41
	exp SYPHILIS/	limit 42 to yr = "1986 – 2006"
	exp TRICHOMONIASIS/	limit 43 to (child <6 to 12 years > or adolescence <13 to 18 years>)
	exp Acquired Immune Deficiency Syndrome/	
	exp Sexual Behavior/	
	(sex$ adj3 behaviour$).tw.	
	(sex$ adj3 transmit$ adj3 (disease$ or infection$)).tw.	
	or/38–47	
	22 and 34 and 37 and 48	
	limit 49 to (school child <7 to 12 years> or adolescent <13 to 17 years>)	

### Study selection

Selection of studies based on agreed *a priori *criteria was done by the first author. To be included for review, studies had to have had an appropriate control group, with participants at post intervention assessment having also been assessed at baseline to increase objectivity in attributing effect(s) of interventions to the original baseline sample, and to increase validity of measured attrition rates. Studies also had to have been conducted in a school setting with youth <19 years (hence excluding studies in institutions of higher learning), in a country in sub-Saharan Africa. Our outcomes of interest included knowledge, attitudes, behavioural intentions and actual behaviour change in prevention of HIV/AIDS and STI.

Data extraction and quality assessment forms were developed specifically for this review (see Additional file 1), based on commonly used forms (from previous systematic review work by the second author) and guided by other reviews on relevant topics and contexts [11,12,19,20], to describe the methodological features of selected studies.

A pilot data extraction and quality assessment was completed on two studies by the first author and double-checked by the second author. All other selected full text papers were subsequently appraised and data extracted by the first author. Differences of opinion on data extraction on the first two studies were resolved by discussion among the first two authors, and where necessary in the entire process of full text appraisal, further clarification was sought from the third author (particularly for the studies that used repeated cross-sectional design [14,17,21,22].

## Results

**Figure 1:**
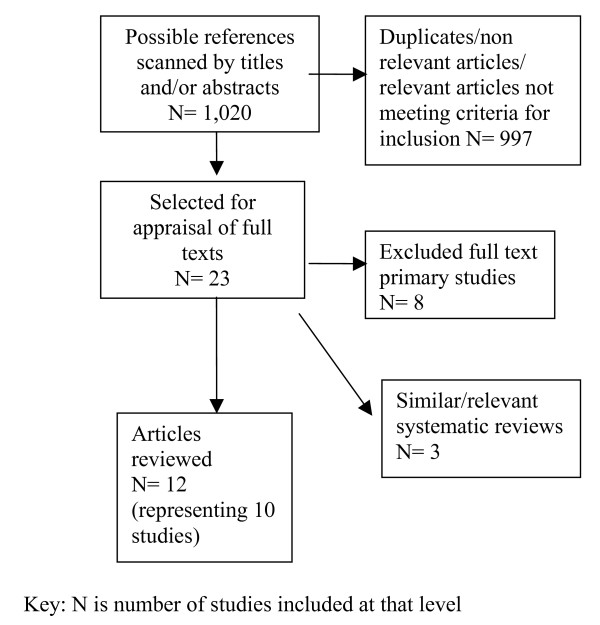
Flow chart for study selection.

Results of search and study selection are shown on the flow chart in Figure 1.

A total of twelve articles (ten studies) met all criteria and have been reviewed and included in this paper.

Table 2 gives the list of studies excluded after full text appraisal and reasons for exclusion, while Table 3 provides details of the included studies.

**Table 2:** List of excluded primary studies and secondary reviews.

**Article details:**	**Ref.**	**Reason(s) for exclusion.**
Brieger WR *et al.*: **West African Youth Initiative: outcome of a reproductive health education program**. *J Adolescent Health *2001, **29**: 436–446.	[14]	Repeated cross-sectional design felt to limit our objectivity in measuring programme attrition.
Gallant M, Maticka-Tyndale E: **School-based HIV prevention programmes for African youth**. *Soc Sci Med *2004, **58**:1337–1351.	[11]	Secondary review, including studies without control group.
Kinsman J *et al.*: **Implementation of a comprehensive AIDS education programme for schools in Masaka District, Uganda**, *AIDS Care*1999, **11**: 591–601.	[35]	No control group
Kinsman J *et al.*: **Evaluation of a comprehensive school-based aids education programme in rural Masaka, Uganda**, *Health Education Res *2001, **16**: 85–100.	[36]	No control group
Maclachlan M *et al.*: **AIDS education for youth through active learning: A school-based approach from Malawi**, *Int J Educ Dev *1997, **17**: 41–50.	[37]	Matched control used. Did not fulfil the criteria of having both pre- and post-intervention assessment.
Magnani R *et al.*: **The impact of life skills education on adolescent sexual risk behaviors in KwaZulu-natal, South Africa**, *J Adolescent Health*, 2005, **36**: 289–304.	[38]	No control group
Magnussen L *et al*.: **Interventions to prevent HIV/AIDS among adolescents in less developed countries: are they effective? ***Int J Adolescent Med Health*, 2004, **16**: 303–323.	[12]	Secondary review, not as focused geographically and in terms of types of intervention.
Mbizvo MT *et al.*: **Effects of a randomized health education intervention on aspects of reproductive health knowledge and reported behaviour among adolescents in Zimbabwe**, *Soc Sci Med *1997, **44**: 573–577.	[39]	Outcomes relating to pregnancy and contraception, and excluding STD/HIV/AIDS.
Okonofua FE *et al*.: **Impact of an intervention to improve treatment-seeking behavior and prevent sexually transmitted diseases among Nigerian youths**, *Int J Infect Dis*, 2003, **7**: 61–73.	[22]	Repeated cross-sectional design felt to limit our objectivity in measuring programme attrition.
Shuey DA *et al.*: **Increased sexual abstinence among in-school adolescents as a result of school health education in Soroti district, Uganda**, *Health Educ Res*, 1999, **14**: 411–419.	[17]	Repeated cross-sectional design felt to limit our objectivity in measuring programme attrition.
Speizer IS *et al.*: **The effectiveness of adolescent reproductive health interventions in developing countries: A review of the evidence**, *J Adolescent Health*, 2003, **33**: 324–348.	[13]	Secondary review, not as focused, geographically/intervention types. Includes studies without control group.

### Description of included studies and participants

Of the ten studies, four were non-randomised [18,23,24]. Rusakaniko *et al*. [18], Fitzgerald *et al. *[25] and Stanton *et al*. [26] were randomised at student or individual level, while others were cluster-randomised to schools. Table 3 shows that eight of the ten studies were conducted in Southern Africa (three in the Republic of South Africa, two in Namibia, two in Zimbabwe and one in Zambia), one in West Africa (Nigeria), and one in Eastern Africa (Tanzania).

**Table 3:** Details of studies included in review

**Reference**	**Sample characteristics**	**Details of intervention**	**Study design and analysis**	**Important observations/considerations**
Agha 2002, Agha and Van Rossem 2004 [9,30]	Secondary (boarding) schools in Lusaka (urban), Zambia; N = 913; N_1 _= 759; mean age = 17.9; social class – 'somewhat wealthier backgrounds'; N_2 _= 416.	*Objective *– to promote safer sex practices; *theoretical basis *– not specified; *time setting *– school hours; *duration *– 1 hour 45 minutes; *main media *– factual information and discussion, drama skits and leaflets; *contents *– transmission of HIV, prevention of HIV, promoting abstinence and condom use; *facilitator *– peers; *control *group-peer led water purification programme	*Study type *– Quasi-experimental longitudinal panel; *(random) allocation *– yes, 3 intervention & 2 control schools; *follow-up assessment *– 1 week and 6 months; *analysis *– multivariate logistic regression.	1) High attrition rate due to the Grade 12 at baseline, having passed out of school by second follow-up. 2) Sexual behaviours not expected to be different at 1 week intervention as report at this time a reflection of pre-intervention practice.
Fawole *et al. *1999 [23]	Mixed-sex senior secondary school in Ibadan, (urban) Nigeria. N = 450; N1 = 433; social class-'poor'.	*Objective *– to evaluate knowledge attitude & sexual risk behaviours of students after a comprehensive health education intervention; *theoretical basis*-not specified; *time setting *– not specified; duration – 6 weeks (2 to 6-hour sessions weekly); *main media *– lectures, films, role plays, stories, songs, debates, essays; *content *– methods of prevention of HIV, demonstration of proper use of condoms; *facilitator *– 1 community physician and 2 trained teachers; *control group *– no programme.	*Study type *– (not stated) controlled longitudinal trial; (*random) allocation *– none, 2 intervention schools, unspecified number of control schools; *follow-up assessment *– 6 months; *analysis *– frequency distribution of variables, Chi^2 ^and ANOVA.	1) School principal approved demonstration of condom use. 2) Positive outcomes, though not statistically significant in condom use and recent history of STD. 3) Participants opinions regarding programme was assessed.
Fitzgerald *et al. *1999 [25]	Grade 9 or 11 secondary school students in Omusati and Caprivi regions of Namibia; N = 515 of which mean age = 17 (range 15–18), median grade = 11; *social class *– 'class standing' = 46%; N_2 _= 452.	*Objective*-to evaluate an intervention-'My Future Is My Choice' (based on one – 'Focus on Kids' that had been successful in reducing adolescent HIV risk in the U.S.) among adolescents residing in Namibia; *theoretical basis *– protective motivation theory; *time setting *– after school hours; *duration *– 7 weeks made of 14 (2-hour) sessions, *main media *– narratives, games, facts and exercises and questions and discussions; *contents*-basic facts about reproductive biology, HIV/AIDS, alcohol, substance abuse and violence within a relationship, communication skills across genders and age differences and a frame work for decision making; *facilitator*-volunteer teacher and out of school youth; *control group *– no programme.	*Study type*-randomised trial; *(random) allocation *– yes, of students from 10 schools; *follow-up assessment *– 6 months; *analysis*-Chi^2 ^and ANOVA.	1) Attrition rate higher (statistically significant) among control youth than intervention youth. 2) Other risky behaviour like alcohol use and weapon carrying assessed.
Harvey *et al. *2000 [4]	Standard 8 secondary school students in 5 districts (4 rural, 1 urban) of KwaZulu Natal, South Africa; N = 1080 of which mean age = 17.6 (range 13–29); *social class *– 'average annual income per household ≤ $150.'	*Objective *– to evaluate effectiveness of a high school drama-in education programme 'DramAide', to increase AIDS awareness; *theoretical basis*-not specified; during school hours; *duration *– unspecified; *main media *– 1^st ^phase of play presentation by DramAide team, 2^nd ^by running drama workshops with students and teachers, and 3^rd ^culminating in an open day of drama, song, dance, poetry and posters all prepared and presented by students; *contents *– 'issues surrounding HIV/AIDS'; *facilitator *– DramAide team of nurses, teachers and actors; *control group *– "booklet intervention schools" students given a 10-page booklet in Zulu about prevention and transmission of HIV/AIDS.	*Study type *– RCT; *(random) allocation *– yes, 7 schools to each of the groups; *follow up assessment *– 6 months; *analysis *– linear regression modelling.	1) Areas of questionable validity and relevance noted by the medical research workers at the Public Health Laboratory Service, U.K relative to questionnaire design. 2) The booklets were written in Zulu whereas questionnaires were administered in English. 3) Difference in intensity, supervision and monitoring of intervention between the two study groups.
James *et al. *2005 [31]	Grade 11 secondary school students in the Midlands (a rural and urban, largely Zulu speaking) district of the Province of KwaZulu-Natal, South Africa. N = 1168; N_1 _= 867 N_2 _= 722; *social class *– not specified.	*Objective *– to test the effects of a systematically developed photo-novella ('Laduma') on knowledge, attitudes, communication and behavioural intentions with respect to sexually transmitted infections(STIs); *theoretical basis *– health promotion and social learning; *time setting *– during school hours; *duration *– approximately 1 hour; *main medium *– print media, a comic called Laduma; *contents *– factual information about STIs, protection against STIs including illustration of correct condom use; f*acilitator *– printed material; *control group*-received normal(not AIDS related school lessons) at time of intervention, then Laduma at end of study.	*Study type*-RCT; *random allocation *– yes, 10 schools to control group and 9 schools to intervention group; *follow-up assessment *– 3 weeks from baseline (time of intervention delivery) and 6 weeks post intervention; *analysis *– Chi^2 ^and logistic regression analysis.	1) Larger percentage of participants spoke Zulu, but intervention was delivered in English. 2) High attrition rate (38.2%), attributed to impending school examination. 3) The time to evaluation of 6 weeks too short to validate measured behaviour change. 4) Bias in reporting of sexual behaviour with only changes in favour of intervention regarding condom use being reported. Change regarding abstinence (seemed opposite to desired outcome) was not reported.
Klepp *et al. *1994, Klepp *et al. *1997 [21, 29]	Grade six and grade seven primary school pupils in Arusha and Kilimanjaro regions (mixed urban and rural) Tanzania. N = 2026; N_1 _= 1785; N_2 _= 814 *social class *– not specified.	*Objective *– to test effects of an HIV/AIDS education programme (NGAO); *theoretical basis *– theory of reasoned action and social learning; *time setting*-during school hours; *duration *– 20 hours over 2–3 months; *main media *– instruction by teachers, crafts(like posters), plays, role plays, poetry, group discussions and theme T-shirt; *contents *– communication about AIDS, transmission and prevention of HIV, abstinence and reduced intention to be sexually active and caring for PWA; *facilitator *– 2 teachers and 1 local health worker per school; *control-group *– no programme during study but to receive 'NGAO' at end of study..	*Study type *– RCT; *random allocation *– yes, 12 control schools and 6 intervention schools; *follow-up assessment*-6 months and 12 months; *analysis *– ANOVA.	1) Attrition higher among control schools (though explained to be incidental), 2) Attrition greater among semi urban than rural schools; catholic than protestants; persons with lower baseline level of AIDS information and persons with subjective norms in favour of becoming sexually active. 3) Positive attitude changes regarding sexual intercourse improved in the intervention group (though not reaching statistical significance). 4) Difference between N_1 _and N_2 _due to initial 7th grade students having passed out.
Kuhn *et al. *1994 [27]	A high (secondary) school in Cape Town (urban), South Africa. N = 567 of which, mean age = 18 years (range 12–30); N_1 _= 482; *social class *– 'economically disadvantaged'	*Objective *– to raise awareness about AIDS; *theoretical basis *– not specified; *time setting*-during and after school hours; *duration *– 2 weeks; *main media *– structured classroom information, role plays, games, structured group work, videos and leaflets; *contents *– 'key AIDS messages', condoms made available; *facilitator *– teachers, assisted by nurses; *control group *– no programme delivered.	*Study type *– pilot study; *random (allocation*) – unclear, 1 intervention and 1 control school, *follow-up assessment *– immediate; *analysis *– Chi^2 ^tests.	1) Programme sometimes led to disruption in normal school activities. 2) Full parental involvement despite taboos. 3) HIV positive individual gave talk to school staff. 4) Condoms were actually made available. 5) Intervention integrated into an already existing 'language curriculum'. 6) Questionnaires were translated into Xhosa (the mother tongue). 7) Some material described as racist.
Munodawafa *et al. *1995 [24]	5 rural secondary forms 2 and 3 (grade 9 and 10 U.S A. equivalent) in Masvingo Province (rural) Zimbabwe. N = 315; N_1 _= 285; *social class *– not specified.	*Objective*-to assess the impact of health instruction on knowledge targeting prevention of STD, AIDS and drug abuse and to assess utility of student nurses as health instructors; *theoretical basis *– not specified; *time setting*-during school hours; *duration *– 7 weeks (2 sessions per week, 40 minutes per session); *main media *– health lessons; *contents *– transmission and prevention and psychosocial issues of STD and AIDS, responsible sexual behaviour and problem-solving and decision-making strategies and others relating to drug abuse; *facilitator *– student nurses; *control group *– no programme delivered.	*Study type *– quasi-experimental; *(random) allocation *– none, 2 schools to control group and 3 to intervention group; *follow-up assessment *– immediate; *medium of assessment *– 'inventories'; *analysis *– ANCOVA and Chi^2^.	1) Study not very focused relative to sexual health intervention as emphasis more towards promoting use of student nurses as facilitator of intervention. Greater part of study evaluation is the perceived performance of the student nurses in intervention delivery. 2) No attempt seems to have been made to validate the questionnaires. Item on 'possibility of getting AIDS by giving blood' actually yielded the reverse of expected outcome. 3) Alcohol, marijuana and tobacco use, part of the content of programme.
Rusakaniko *et al. *1997 [18]	All types of secondary schools (boarding and day schools, mixed- & single-sex schools) in rural & urban areas Zimbabwe. N = 1673; N_1 _= 1568 and N_2 _= 1589; *school classes *– forms 1 to 6; *social class *– unspecified.	Objective- to determine the impact of an intervention package on knowledge of various health issues; theoretical basis- not specified; time setting-not specified; duration- not specified; main media-lectures, videos, and leaflets and pamphlets; *contents*-reproductive biology, STD and AIDS, responsible sexual behaviour and unwanted pregnancy and contraception; *facilitator*-teachers and printed material; *control group *– No programme delivered.	*Study type *– (wrongly described as an RCT); *(random) allocation*-none, purposive sampling of schools and random sampling of students; *follow-up assessment *– 5 months and 7 months; *analysis*-linear trend and Chi^2 ^test.	1) Stated to be RCT, had random sampling rather than randomisation. 2) Religious bias noted. 3) Not quite focused in terms of study population. Though schools were stratified for representatives, results are analysed as if study sample is homogenous. 4) Some documented 'decrease in knowledge level' following intervention in relation to HIV/AIDS transmission and pregnancy 7) Aims and objectives not clear throughout the study.
Stanton *et al. *1998 [26]	10 secondary schools in Caprivi and Omusati districts of Namibia. N = 515; median age = 17 and median grade= 11; N_1 _= 452; N_2 _= 379; N_3 _= 359; *social class *– not specified.	*Objective*- to evaluate HIV risk-reduction intervention 'My Future is My Choice', based on 'Focus on Kids' curriculum developed for African-American youth in US; *theoretical basis *– social cognitive theory; *time setting *– after school hours; *duration *– 7 weeks (14 × 2-hourly sessions); *main media *– narratives, games, facts & exercises, questions & discussion; *contents *– basic facts about reproductive biology, HIV/AIDS and other risk behaviours includ. alcohol, intra-relationship violence, communication skills and framework for decision-making; *facilitator *– volunteer teacher & out-of-school youth; *control group *– delayed-control condition.	*Study type *– RCT; *(random) allocation *– yes of individuals; *follow-up assessment *– immediate, 6 months and 12 months; *analysis *– Chi ^2 ^analysis.	1) Attrition rate significantly higher in control group, older respondents, and in Caprivi region.

Key: N = No. at baseline; N_1 _= No. at 1^st ^assessment; N_2 _= No. at 2^nd ^assessment; N_3 _= No. at 3^rd ^assessment.

Even though the target group for our review was adolescents (hence our exclusion of higher education institutions), Kuhn *et al. *[27] and Harvey *et al. *[28] reported maximum ages of 29 and 30 years respectively. Two studies reported mean ages of 13.5 years. The first [21,29] was conducted in a primary school and the second [18] assessed age at menarche (age of students at the time of evaluation was not stated). The reported mean (or median) age in all other studies was 17 years.

Only one study was conducted in a primary school [21,29]. Agha and Van Rossem [30] conducted their study in secondary boarding schools, whilst Rusakaniko *et al. *[18] had a mix of boarding, day, single sex and co-educational secondary schools. All other studies were conducted in secondary day schools.

Reported socio-economic status of participants was above average in one study [9,30] and poor in three studies [23,27,28]. No other studies described the socio-economic status of participants. The level of development of the larger communities where schools were located was rural in Munodawafa *et al*. [24], urban in Fawole *et al*. [23] and Kuhn *et al*. [27], while all others studies' schools were located in mixed rural and urban communities.

Sample sizes at baseline ranged from 315 [24] to 2,026 [21] while attrition rates ranged from 3.8% (at six months follow-up) [23] to 38.2% (at nine weeks follow-up) [31], a reflection of the high rates of attrition and school absenteeism common in African countries [32].

A 'no intervention' alternative was applied to all but the following three studies. In James *et al. *[31], the control group had normal school lessons while print media was being read by the intervention group. Harvey *et al*. [28] compared a booklet intervention in the control group to an intense intervention utilising drama activities with key AIDS themes for the intervention group. Agha and Van Rossem used the same facilitator (peers) but different themes (water purification for the control group and sexual education for the intervention group) [9,30].

### Description of interventions

Theoretical basis for the interventions were explicitly stated in four studies only [25,26,29,31]. All but two studies [18,24] clearly expressed an active student participatory component of interventions such as involvement in discussion, exercises, or organising school events with an AIDS theme.

In seven of the ten studies, a combination of activities was employed in the delivery of the intervention (e.g. giving factual information, employing active participatory element like drama, songs and craft, and holding discussion or question and answer forums). Other studies employed a single medium each, such as printed material only in James *et al. *[31], classroom lessons only in Munodawafa *et al. *[24], and comparison of printed material only (control group) to drama only (intervention group) in Harvey *et al*. [28]. In Harvey *et al. *[28], reported programme intensity, length and monitoring appeared greater in the drama group than the control one.

Programme facilitators were peers in Agha and Van Rossem [9,30], health professionals in Munodawafa *et al*. [24], a teacher and some other facilitator in all remaining studies except James *et al*. [31], who used printed material only.

The interventions were delivered after school hours in studies by Fitzgerald *et al*. [25] and Stanton *et al. *[26]. Kuhn *et al. *[27] delivered the intervention both during and after school hours. In all other studies but two which did not specify [18,23] the intervention was delivered during school hours.

All studies' messages included general facts about transmission and prevention of HIV and abstinence promotion. Two studies actively included some form of demonstration or description of condom use [23,31]. In Kuhn *et al*. [27] condoms were made available, but the authors reported that students still had to overcome their perceived barriers to requesting condoms from a vocational teacher.

For all other studies, condom use was either explicitly stated to be part of the intervention messages [28,30], or could be inferred from the fact that they constituted part of the outcomes measured [24,26] or that there was a contraception component included in the intervention [18]. Klepp *et al*. [29] reported that teachers avoided the condom use messages despite the educational policy permitting condom-use messages as part of school sexual health interventions at the time the study was evaluated.

The peer-led intervention by Agha and Van Rossem [30] was a single one hour and 45 minute session for the intervention group (one hour for controls). Average reading time for the print-only intervention by James *et al*. [31] was about one hour. Two studies did not report the duration of the intervention [18,28] but the remaining studies' interventions were delivered over a period of weeks (usually some hours or sessions each week).

### Description of outcomes

All studies conducted assessments using self-administered questionnaires in English. Results of outcomes, grouped under themes and sub-themes are shown in Table 4.

**Table 4:** Results of Outcomes

**Outcomes**\**27 Reference**	Agha 2002, Agha, Van Rossem 2004 [9,30]	Fawole et al. 1999 [23]	Fitzgerald et al. 1999 [25]	Harvey et al. 2000 [4]	James et al. 2005 [31]	Klepp et al. 1994, Klepp et al. 1997 [21,29]	Kuhn, et al. 1994 [27]	Munodawafa et al. 1995 [24]	Rusakaniko et al. 1997 [18]	Stanton et al. 1998 [26]
General STD/HIV/AIDS knowledge	**+/+**	**+**	**+**	**+**	**+**	**+**	**+**	**+**	**0**	
Knowledge regarding to abstinence	**+/+**									
Knowledge regarding condom use	**+/-**						**+**	**+**		
Attitudes towards PLWHA					**+**	**+**	**+**			
Attitude towards abstinence			**+**							
Attitudes towards condom use			**+**		**+(m)**					
Attitudes towards sexual intercourse			**+**			**0**				
General risk perception AIDS/STD/HIV	**0/?-**	**+**		**+**						
Intentions regarding abstinence			**+**		**?-**	**+**				
Intentions regarding condom use			**+**		**+**		**0**			**+/0/0(f)**
General sexual behaviour assessment				**0**						
Multiple Sexual partnerships	**0/+**	**+**								
Behaviour regarding abstinence		**+**	**0**	**0**		**0**				**0/0/+(f, bv)**
Behaviour regarding condom use		**0**	**0**	**+**	**0**					**+/0/0(m, bv)**
Symptom of STD		**0**		**0**						
Communication with others on HIV/AIDS					**0**		**+**			**0/+/0(m)**

**Key**:

0 No statistically significant effect in direction of desired outcome

- Reversal of effect or effect in opposite direction to desired outcome

+ Statistically significant effect in direction of desired outcome

/ Earlier follow-up result to left of, and latter follow-up effect to right of

f effect significant in females only

m effect significant in males only

bv observed effect significant among baseline virgins

? hint of effect reported to the right of this symbol

Studies were judged to be heterogeneous in terms of outcomes measured, time to evaluation, and statistical analyses employed. For example, one of four questions under our sub-theme, 'attitudes to persons with AIDS' was framed as "I would visit a friend if I knew he had the AIDS virus" and evaluated at six months and twelve months post intervention by Klepp *et al. *[29]. We grouped under the same sub-theme the single item by Kuhn *et al*. [27], "would accept someone with AIDS into their class" which was evaluated in the immediate post-intervention period.

**General knowledge regarding HIV/AIDS/STD **was evaluated in all but one study [26]. All other studies, except Rusakaniko *et al*. [18] which was not very focused in terms of types of schools, contents of intervention and outcomes evaluated, reported significant positive results in the desired direction.

**Knowledge and normative beliefs regarding abstinence **evaluated by Agha and Van Rossem [9,30] were significantly improved both at immediate post-intervention, and at the six-month follow-up.

**Knowledge regarding condoms **was evaluated immediately following the intervention in three studies [9,24,27] with significant effects, but in the six-month follow up assessment later conducted by Agha and Van Rossem [30], this change had disappeared, suggestive of a decay effect of the intervention.

**Changes in general attitude regarding AIDS **such as 'seeing HIV/AIDS as everyone's problem' was evaluated with significant desired effects by Harvey *et al. *[28] and Fawole *et al. *[23]. Personal risk perception regarding AIDS evaluated by Agha and Van Rossem [9,30] yielded no immediate effect at baseline, and a hint of actually going in a direction opposite to the desired outcome at the six-month evaluation.

**Attitudes to persons living with HIV/AIDS (PLWHA) **improved in all three studies where evaluated [27,29,31]. Further sub group analysis done by James *et al*. [31] showed that the significant effect observed was among males only.

In Fitzgerald *et al*. [25]** attitudes regarding abstinence **was assessed with statements like 'being intimate without having sex', and 'being friends for a long time without having sex' with statistically significant desired effect of the intervention.

Assessment of **attitudes towards condom use **at six months in Fitzgerald *et al*. [25] and six weeks in James *et al*. [31] yielded significant positive outcomes, but no significant effect was reported by Klepp *et al*. [29] who reported outcomes at one year follow-up. We are unable to conclude that the intervention was only successful in the short-term since the facilitators in Klepp *et al*. [29] avoided the condom use message. Stanton *et al*. [26] also evaluated perceived self efficacy regarding condom use with significant positive outcomes at six months and 12 months.

**Intentions regarding abstinence **evaluated by Fitzgerald *et al. *[25] and Klepp *et al*. [29] showed significant desired effects. In James *et al. *[31], there was a significantly greater percentage of control youth reporting intentions to abstain than intervention youth. This finding in James *et al*. [31] appeared due to abstinence and intentions to use condoms being analysed as mutually exclusive outcomes rather than independent (there was a significantly greater percentage of intervention than control youth reporting intentions to use condoms). Further sub-analysis by James and colleagues [31] of future intentions regarding abstinence showed significant improvement among females and among baseline virgins.

**Intentions regarding condom use **were evaluated in four studies. Desired outcomes reached statistical significance in Fitzgerald *et al*. [25] at six months and James *et al. *[31] at both the three-week and six-week follow-up. In James *et al*. [31], further sub-group analysis showed that intentions to use condoms was positively predicted by sex being male and persons who had been sexually active at baseline. Kuhn *et al. *[27] reported no effect immediately post-intervention on intentions to use condoms but in Stanton *et al. *[26], the significant change in intentions observed immediately post intervention had worn off by the six-month and 12-month follow-up assessments.

**Change in number of sexual partners **evaluated by Fawole *et al*. [23] at six months post intervention was associated with significant positive outcomes. In Agha and Van Rossem [9,30], assessment at immediate post-intervention showed no significant effect (as could be expected since sexual behaviours at this time were still a reflection of pre-intervention practice) but significant effect of change was reported at the six-month follow-up.

**Increase in reported practice of abstinence **following intervention at six-month follow up was significantly more in intervention than control group in Fawole *et al*. [23], but did not reach levels of statistical significance in three studies [25,28,29]. In Stanton *et al. *[26], the practice of abstinence was not significantly improved until the 12-month follow-up and only among females and baseline virgins.

**Positive outcomes regarding actual condom use **was evaluated in five studies. In four of these [23,26,28,31], there was no significant change except for an immediate post-intervention significant effect observed in a subset made of males who were baseline virgins in Stanton *et al. *[26]. Although there were no statistically significant changes within the intervention group in the fifth study [25], significant inter-group differences were reported on condom use at the six-month follow-up due to a drop in the percentage of youth using condoms in the control group.

Improvement in **clinical outcome **(recent history or symptoms of any STI) assessed by Fawole *et al*. [23] and Harvey *et al*. [28] did not reach statistically significant levels.

**Communication with new sexual partners **about past sexual experiences was evaluated by Stanton *et al. *[26] and significant improvement was observed in males at the six-month evaluation, but had worn off at the time of the 12-month follow-up assessment.

**General communication**, i.e. not necessarily with sexual partners, on HIV/AIDS was very significantly improved in Kuhn *et al. *[27] where parental consent was sought very early in intervention design, and in Klepp *et al*. [29] but not in James *et al. *[31].

## Discussion

This review found a paucity of published school-based interventions in sub-Saharan Africa relative to the magnitude of the AIDS epidemic. Participants' socio-demographic characteristics such as age, sex and school grade were generally well reported in our included studies, but socio-economic status and religion were less well reported. Description of socioeconomic status and religious affiliation in future studies could better inform on programme generalisability to other settings.

Justification of sample size and validation of measurement tools were not reported in many studies. Although no studies justified the timing of intervention delivery, conducting the intervention during school hours seemed to give the opportunity for more interaction between researchers and stakeholders in Kuhn *et al. *[27] even though the programme reportedly led to "a disruption in normal school activities". Future studies should address both these preceding aspects of the intervention, and others relating to establishing the context for the particular intervention delivered such as duration and programme monitoring as discussed below.

To enable facilitators to effectively conduct the intervention and its monitoring training should be provided rather than assuming facilitators' knowledge and perceptions on the particular sexual health intervention under evaluation. For example, Klepp *et al. *[29] reported that teachers avoided condom-use messages despite supportive policies being in place at the time of the evaluation, and this was only acknowledged after delivery of the intervention had been concluded. Future studies should be conducted after establishing an appropriate framework, i.e. one informed by both theory and research evidence from systematic reviews, qualitative studies and discrete choice experiments (which may ascertain participants preferences and justify the intervention and components or methods employed in its evaluation). This is recommended for the evaluation of complex interventions [33], of which prevention HIV/AIDS among adolescents is representative. If training of staff, a theoretical basis and evidence-based interventions had been part of the studies in our systematic review it could have helped explanation why behavioural change has been so difficult to achieve in HIV/AIDS prevention programmes among Sub-Saharan African adolescents.

Tactical communication of the goals of an intervention, to avoid areas or terms that lead to unending debates, is a characteristic that helps in smooth delivery of interventions. In Kuhn *et al*. [27] for instance, the intervention was portrayed as 'a means to prevent STD/HIV/AIDS' rather than as 'providing sex education' to stakeholders, making it more acceptable. Such involvement of stakeholders, particularly parents and teachers, very early in the design of the intervention, and exhibiting cultural sensitivity in a community where it had hitherto been a taboo for adults and pupils to discuss sexual issues, could have led to the significant improvement in communication between youths and their parents and teachers [27]. Fawole *et al*. [23] reported that the school principal gave full support to the condom content of the intervention, also following involvement in the intervention design.

Use of multiple media and activities in communication or delivery of messages was employed in almost all studies. More than one facilitator was also used in many studies but we are unable to determine the relative contribution of each medium or facilitator to programme effectiveness within the scope of this review. However, the effectiveness of the different interventions appeared similar across all studies from the overall trend of effectiveness by reported significant change as shown in Table 4. More focused studies or studies employing factorial designs to vary different programme components could further inform on the strengths and weaknesses of using different media and facilitators in intervention design and delivery.

Skill-based content of interventions involving active participation of students and more lengthy interventions offering the opportunity for repeated exposure to the same theme appeared to be associated with greater effectiveness. This was exemplified by comparing the 'DramAide' intervention by Harvey *et al*. [28] which was longer and employed intense involvement of students compared to the 'booklet intervention' control group.

Many studies reported outcomes at immediate post-intervention or short term (≤ six months) follow up. To reduce limitation in the long-term evidence of effect of school-based interventions to prevent STI/HIV in sub-Saharan Africa and to determine if certain outcomes routinely demonstrate a delay effect (e.g. reported change in practicing abstinence did not reach statistical significance until 12 months in Stanton *et al*. [26], future studies should be designed to be able to report long-term (≥ one year) outcomes.

Design of future interventions in terms of focus is important. Rusakaniko *et al*. [18] which was not very focused in terms of types of schools, content of intervention and outcomes measured reported no significant change, not even in knowledge at the end of the intervention. Conducting more focused interventions could be more informative in making associations to both internal and external validity of a study.

Employing sub-analysis of outcomes based on pre-intervention sexual history as conducted by Stanton *et al*. [26] and James *et al*. [31] is highly recommended for future research towards the development of interventions that are more tailored to fit specific groups.

Certain attributes of effective sexual health interventions have been described to include having clear and focused outcome behaviour, addressing all social influences relating to the students, including opportunity for practice of communication and negotiation skills, involving students in experiential activities towards personalising relevant information, having their foundation in social learning theories and having their content designed to reinforce group specific values and norms [34]. The findings of our review are not in contrast with any of these. There was also similarity in the trends for effectiveness of interventions in our included studies, in effecting change in knowledge, attitudes, and behaviour change, when compared to a previous review by Gallant and Maticka-Tyndale [11]. To summarise the effectiveness of interventions in the population represented within the scope of this review, we found that the most significant changes were reported in knowledge, being followed by changes in attitudes. Outcomes relating to future intentions were next, while the least significant changes were in actual behaviour.

Although our aim was to conduct a focused review with good quality studies that would be valid and generalisable to sub-Saharan Africa, we acknowledge that this review is limited by the possibility of having missed relevant studies within the timescale and resources available. Although we applied no language limitations to our searches, all our studies were conducted and/or reported in English, implying the possibility of having missed relevant studies conducted in non-English speaking African countries. Most of our studies were conducted in Southern Africa and our strict exclusion criteria led to the exclusion of some studies, mostly from Eastern Africa, which might limit the generalisability of our findings to all sub-Saharan Africa.

## Conclusion

This review acknowledges the great need for interventions aimed at reducing STI/HIV/AIDS in a sub-Saharan African context, targeting adolescent still in school education. From the 12 articles (representing 10 studies) reviewed, we concluded that it is relatively easy to effect changes in knowledge and attitudes regarding STI/HIV/AIDS using school-based interventions that have been carefully designed to suit the sub-Saharan Africa environment. It is more challenging to effect changes in positive intentions regarding sexual risk reduction, and most of all, changes regarding sexual risk behaviours. Measured changes in behaviour either did not reach statistically significant levels, or when they did in the immediate post-intervention period, wore off within weeks to months. Some behaviour changes however appeared to exhibit a delay effect in development. Behavioural change in relation to abstinence was easier to effect among baseline virgins, while condom use appeared to be the more practicable sexual risk protective behaviour for adolescents who are already sexually active.

The magnitude of the HIV epidemic, and current evidence of relative lack of sexual health interventions targeting young people in sub-Saharan Africa calls for more research and scrupulous use of available resources to inform the design and delivery of well-tailored interventions to meet the unique needs of this population group. Future studies employing a more systematic approach, conducted after an established contextual framework for the intervention is determined [33] are urgently needed to help halt and possibly reverse the course of the AIDS pandemic.

## Competing interests

The author(s) declare that they have no competing interests.

## Authors' contributions

VAP-E, AP and EvT have contributed to the original design of the systematic review. VAP-E developed the research protocol for the review, and VAP-E and EvT established the a priori criteria for the studies. VAP-E and AP developed the search strategy, VAP-E and AP piloted the data extraction and quality assessment. VAP-E appraised and extracted the data from the full text papers. All authors were involved in the writing of the paper, and all approved the final submission.

## Pre-publication history

The pre-publication history for this paper can be accessed here:



## Supplementary Material

Additional file 1 **Data Extraction and Quality Assessment Forms for Studies**. This additional shows the forms we have used for (1) data extraction and (2) quality assessment for the included studies. Fellow researchers may find this form of use for future systematic reviews. Click here for file
