# Supplementary material for: A systematic review of school-based sexual health interventions to prevent STI/HIV in sub-Saharan Africa
Source: BMC Public Health. 2008 Jan 7;8:4. doi: 10.1186/1471-2458-8-4 (PMC2248569; doi:10.1186/1471-2458-8-4)
Supplement: Additional file 1 — Data Extraction and Quality Assessment Forms for Studies. This additional shows the forms we have used for (1) data extraction and (2) quality assessment for the included studies. Fellow researchers may find this form of use for future systematic reviews. [file 1471-2458-8-4-S1.doc]

**Data Extraction and Quality Assessment Forms for Studies**

Systematic Review of Effectiveness of School-Based Sexual Health Interventions in Sub-Saharan Africa.

**Bibliographic Details**

Authors …………………………………………………………………..

Journal ……………………………………………………………………

Year ………… Volume ……….. Issue…………….Pages……………………….

Title:………………………………………………………………………………………………………………………………………………………………………………………………………………………………………………………………………………………

Country………………………………

Database………………………………….. Reference I.D

Others (e.g. reference checking) ………………………………………………………….

**Eligibility check**

|  | Yes | No | Unclear or other with details |
| --- | --- | --- | --- |
| Study with control group prospectively |  |  |  |
| School-based setting of intervention |  |  |  |
| Outcome related to prevention of HIV/AIDS/STD |  |  |  |
| At least one pre- and one post- intervention measure. |  |  |  |

**Study characteristics**

Study design:

Name of intervention:

Theoretical basis:

Objective(s):

Details –randomisation: yes/ no

-control group(s)

-intervention group(s)

Sample characteristics at baseline

|  | Control | Intervention |
| --- | --- | --- |
| Number of participants-Male:  Female:  Total: |  |  |
| Age of participants |  |  |
| School grade/level |  |  |
| Social class |  |  |
| Location(urban/rural) |  |  |
| Faith/religion |  |  |
| Others |  |  |

**Detail of interventions**.

|  | Control | Intervention |
| --- | --- | --- |
| Setting |  |  |
| Duration and frequency |  |  |
| Main activities  (medium) |  |  |
| Content |  |  |
| Instructor |  |  |
| Instructor Training |  |  |
| Programme monitoring |  |  |
| Other issues  (e.g. resistance) |  |  |

Follow-up/assessment

| Number of follow-ups |  |
| --- | --- |
| Time(s) to evaluation. |  |
| Medium of assessment |  |
| Non-response/ attrition details |  |
| Outcomes measured(list) |  |
| Other issues arising(e.g. adverse effect): |  |

**Results/Measurement of intervention effect.**

Analytic method-

Changes in outcomes-

**Quality assessment form**

|  | Yes | Uncertain /Implied | No/ |
| --- | --- | --- | --- |
| 1. clear objective(s) | 2 | 1 | 0 |
| 2. sample size justified | 2 | 1 | 0 |
| 3.sampling method justified | 2 | 1 | 0 |
| 4. contamination of groups | 1 | 0 | 2 |
| 5. facilitator training | 2 | 1 | 0 |
| 6.programme monitoring | 2 | 1 | 0 |
| 7.validated outcome measures | 2 | 1 | 0 |
| 8.groups comparable at baseline | 2 | 1 | 0 |
| 9.untoward events occurring in study | 1 | 0 | 2 |
| 10.relevant outcomes considered | 2 | 1 | 0 |
| 11. reliability of measurement tools | 2 | 1 | 0 |
| 12. adequate follow up | 2 | 1 | 0 |
| 13. drop-out details described | 2 | 1 | 0 |
| 14. basic data described adequately | 2 | 1 | 0 |
| 15.appropriate statistical tests | 2 | 1 | 0 |
| 16.uncertainties with time and others considered | 2 | 1 | 0 |
| 17.assessment of statistical significance | 2 | 1 | 0 |
| 18. objective interpretation of main findings | 2 | 1 | 0 |
| 19.negative findings interpreted | 2 | 1 | 0 |
| 20. limitations stated and accounted for | 2 | 1 | 0 |

Maximum possible score 2x20…………………………

Overall rating = total score expressed as a percentage of maximum score.

Weak = < 50%

Moderate = 51-80%

Strong =81-100%.
